# The effects of urban land use gradients on wild bee microbiomes

**DOI:** 10.3389/fmicb.2022.992660

**Published:** 2022-11-17

**Authors:** Phuong N. Nguyen, Sandra M. Rehan

**Affiliations:** Department of Biology, York University, Toronto, ON, Canada

**Keywords:** bacteria, fungi, urbanization, microbial diversity, land use, Apidae, Ceratina

## Abstract

Bees and their microbes interact in complex networks in which bees form symbiotic relationships with their bacteria and fungi. Microbial composition and abundance affect bee health through nutrition, immunity, and fitness. In ever-expanding urban landscapes, land use development changes bee habitats and floral resource availability, thus altering the sources of microbes that wild bees need to establish their microbiome. Here, we implement metabarcoding of the bacterial 16S and fungal ITS regions to characterize the diversity and composition of the microbiome in 58 small carpenter bees, *Ceratina calcarata,* across urban land use gradients (study area 6,425 km^2^). By categorizing land use development, green space, precipitation, and temperature variables as indicators of habitat across the city, we found that land use variables can predict microbial diversity. Microbial composition was also found to vary across urban land use gradients, with certain microbes such as *Acinetobacter* and *Apilactobacillus* overrepresented in less urban locations and *Penicillium* more abundant in developed areas. Environmental features may also lead to differences in microbe interactions, as co-occurrences between bacteria and fungi varied across percent land use development, exemplified by the correlation between *Methylobacterium* and *Sphingomonas* being more prevalent in areas of higher urban development. Surrounding landscapes change the microbial landscape in wild bees and alter the relationships they have with their microbiome. As such, urban centres should consider the impact of growing cities on their pollinators’ health and protect wild bees from the effects of anthropogenic activities.

## Introduction

Research on bee microbiomes uncovered their vital role in many aspects of bee health, including improving immunity (Engel et al., 2012; Mockler et al., 2018; Rubanov et al., 2019), nutrient utilization (Engel et al., 2012; Raymann and Moran, 2018), and reducing metalloid toxicity (Rothman et al., 2019). For example, the presence of non-pathogenic microbial symbionts in honey bees upregulates antimicrobial peptides in the bee and prepares the immune response against pathogenic microbes (Kwong et al., 2017a). The importance of microbes can also be implicated more broadly, with the sterilization of larval mason bee microbiomes negatively impacting bee fitness through declining growth rates, biomass, and survivorship (Dharampal et al., 2019). The gut microbiome has even been associated with different behavioural tasks in honey bees and memory retention in bumble bees (Jones et al., 2018; Li et al., 2021). With the common consensus that microbes engage in beneficial interactions with their bee hosts, research continues to examine the factors that influence the establishment and maintenance of the microbiome.

The core microbiome is described as the microbes that are consistently found within many individuals of a species (Turnbaugh and Gordon, 2009; Danko et al., 2021). Social and solitary bees acquire their microbiome in different ways, with honey and bumble bee microbiome composition directly influenced by social interactions with their colony members (Martinson et al., 2012; Tarpy et al., 2015; Kwong and Moran, 2016; Elijah Powell et al., 2018; Su et al., 2021), whereas less social wild bees inherit their microbiome from their surrounding environment and from their diet (McFrederick and Rehan, 2016; Dew et al., 2020; Voulgari-Kokota et al., 2020; Figueroa et al., 2021; Kapheim et al., 2021). Much work has been done on social bees such as *Apis* and *Bombus* to characterize and examine the health effects of an altered core microbiome (Martinson et al., 2011; Raymann and Moran, 2018; Rothman et al., 2019; Su et al., 2021). For solitary wild bees, this research is in its infancy; however, it is known that wild bees do not always maintain the same consistent core microbiome seen in social bees (McFrederick et al., 2012, 2014; Kwong and Moran, 2016; McFrederick and Rehan, 2016; Graystock et al., 2017). An example on how solitary wild bees can have differing core microbiomes can be found in *C. calcarata*, a wild bee displaying different core microbiomes from other bee species and across different regions (Graystock et al., 2017; Dew et al., 2020; Nguyen and Rehan, 2022; Shell and Rehan, 2022).

A range of variables, including developmental stage (McFrederick et al., 2014; Kapheim et al., 2021; Nguyen and Rehan, 2022), sociality of host species (Mohr and Tebbe, 2006; McFrederick et al., 2014; Graystock et al., 2017), climate (McFrederick and Rehan, 2019), geographical location (Almeida et al., 2022), and landscape features (Cohen et al., 2020), have been examined to determine their effects on the microbiome. Examining microbiomes across host developmental stages has allowed for closer examinations of the establishment of microbes, showing that diet is the main source of bacteria and fungi for developing solitary bees (Dew et al., 2020; Pozo et al., 2020; Kapheim et al., 2021; Nguyen and Rehan, 2022). Sociality can influence the bee microbiome by impacting how solitary and social bees interact with food resources, the environment, and other bees to transmit different microbes (Mohr and Tebbe, 2006). Across various bee species, environmental factors such as climate (McFrederick and Rehan, 2019), agriculture (Motta et al., 2018; Muñoz-Colmenero et al., 2020), natural habitat, floral resources, and wild bee diversity in the landscape can all shape microbe composition (Cohen et al., 2020; Shell and Rehan, 2022). In previous studies of *Osmia lignaria,* the presence of some microbes, such as *Apilactobacillus* sp., was associated with increased green spaces and an increased relative rarified ASV abundance in bees from less developed landscapes as opposed to urban and highly developed sites (Cohen et al., 2020). Thus, microbial members within the microbiome are subject to many different factors that can change their abundance, composition, and diversity.

The impact of a changing environment on bees and their microbiome needs to be studied as urbanization and anthropogenic activities continue to alter bee habitats in growing cities (Ritchie and Roser, 2018; Wilson and Jamieson, 2019; Ayers and Rehan, 2021; Prendergast et al., 2022). With more than half of the world currently living in urban areas and projections predicting this number to increase to two thirds of the global population living in a city centre by 2050 (Ritchie and Roser, 2018), the growth of large, developed areas is undeniable. Decreased availability of green space and the urban heat island effect tends to result in increased temperatures and reduced precipitation in these areas (Rinner and Ussain, 2011; Steensen et al., 2022). Urbanization affects the availability of green space, abundance and richness of floral resources, microclimate, and habitat quality for bees, changing the landscape features that can shape bee microbiomes (Goulson et al., 2015; Buchholz et al., 2020; Cohen et al., 2020, 2022; Ayers and Rehan, 2021; Danko et al., 2021). Floral abundances and garden sizes have a direct, positive effect on parasite and pathogen richness that is harmful to bumble bees, attributable to increased transmission from more resource provisioning (Cohen et al., 2022). Wild bumble bees have also been shown to harbour pesticides in both agricultural and urban landscapes (Botías et al., 2017), potentially jeopardizing microbial composition (Kakumanu et al., 2016; Rothman et al., 2020). Characterizing the microbiome of urban bees and how its composition and diversity varies across different landscapes offers an essential step towards understanding contributing factors to changes in bee health.

This study examines the small carpenter bee *C. calcarata* Robertson (Hymenoptera: Apidae). These subsocial bees nest within pithy stems, laying eggs on mass provisions that will provide brood the total nutrition required until they are fully grown (Michener, 2007; Rehan and Richards, 2010). Numerous studies have characterized diversity and composition of the microbiome and pollen provisions in *C. calcarata* (McFrederick and Rehan, 2016; Graystock et al., 2017; Dew et al., 2020; Nguyen and Rehan, 2022). In adult bees this core microbiome consists of 13 bacterial phylotypes, including *Lactobacillus, Acinetobacter, Methylobacterium, Pseudomonas,* and *Gilliamella* (Graystock et al., 2017; Shell and Rehan, 2022), several of which are common in other bee microbiomes as well (Vásquez et al., 2012; Voulgari-Kokota et al., 2019; Kapheim et al., 2021). The *C. calcarata* fungal microbiome includes members such as *Alternaria, Ascosphaera* and *Penicillium* (Nguyen and Rehan, 2022). However, despite various characterizations of this small carpenter bee bacterial and fungal microbiome, closer investigations into the specific factors driving differences in microbial composition and diversity, as well as the functional role of different microbial taxa on maintaining bee health, are fundamental.

The aim of this study is to determine whether the microbiome of adult *C. calcarata* differs across an urbanization gradient including local environmental features: percent land use development, percent green space, temperature, and precipitation. Using 16S and ITS metabarcoding, we examined the respective bacterial and fungal composition and diversity within 58 female small carpenter bees collected across a densely urban landscape, with different levels of urbanization. Here, we hypothesize that bees living under different environmental conditions across an urban land use gradient will result in varying microbial composition. We predict lower microbial diversity and the underrepresentation of beneficial microbes in more urban and developed areas with less available green space, increased temperatures, and reduced precipitation. This research aims to understand the differences in the microbiomes of wild bees living under different levels of urbanization.

## Materials and methods

**Figure 1:**
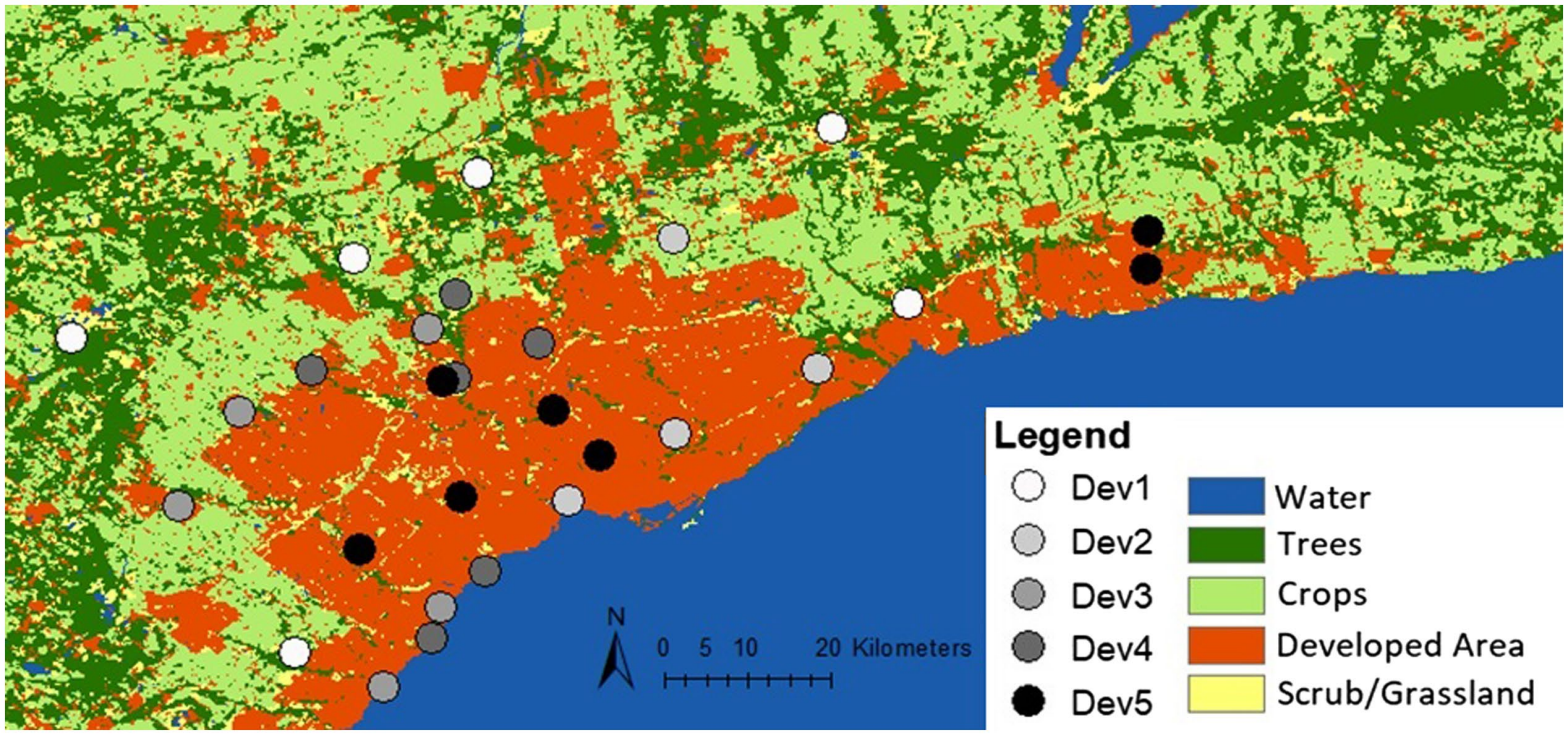
Development distribution and collection locations in and around Toronto, Canada using the Sentinel-2, 10 m land use time series from 2017 to 2021 by ESRI. Circles represent 29 collection sites. The trees class includes trees and flooded vegetation. Crops include human planted grass and crops below tree height. Scrub/grassland consists of bare ground and rangeland, or open areas with homogenous grasses. Developed areas are built areas with human made structures, roads, and impervious surfaces.

In June–July 2019 and 2020, 58 female individuals of *C. calcarata* Robertson (Hymenoptera: Apidae) were collected across 29 sites within Toronto, Canada (43.6532° N, 79.3832° W) (Figure 1). Between one and three bees were selected from each site and sites were chosen to cover a widespread area across the city. *Ceratina calcarata* is a native small carpenter bee commonly found in urban and rural contexts across eastern North America, including within the city of Toronto (Packer et al., 2007; City of Toronto, 2016; Shell and Rehan, 2016; Dew et al., 2020; Kelemen and Rehan, 2021). Nests established in the pithy stems of sumac, *Rhus typhina,* were opened with the lone adult female being removed from each collected nest, flash frozen in liquid nitrogen, and stored at-80°C until DNA extractions.

The collection map was created using the Sentinel-2 land use/land cover timeseries from 2017 to 2021 by Impact Observatory, Microsoft and Esri at a resolution of 10 m (Karra et al., 2021; Figure 1). Collection sites were characterized into five different levels of urban intensity using measurements of the developmental percent, percent green space, mean annual temperature, and annual precipitation (Supplementary Table S1). These categories were assigned by evenly dividing the range of values for each environmental variable into five categories ranging from 1 (very low) to 5 (very high). Landscape features of developmental percent and percent green space were calculated at each collection site using the Ontario Land Cover Compilation (OLCC) v.2.0 in ArcGIS as a percentage of landscape cover within a 500 m radius from the collection point (Land Information Ontario, 2019). Climate data, including mean annual temperature and annual precipitation, were calculated using the same process with WorldClim v.2.0 data at a resolution of 30 s (Fick and Hijmans, 2017). These features provide an overall characterization of urban land use gradients in the study region and were divided into the five categorical levels for later analyses (Supplementary Table S1).

DNA extractions were performed using the Omega-Biotek E.Z.N.A. Soil DNA kit, following the manufacturer’s protocol for 100–250 mg samples, with some modifications as described in Nguyen and Rehan (2022). This included the addition of 100 μg of 1xPBS, 30 μl of proteinase K, 5 μl of RNAse and manual crushing of the bees using a sterile pestle. DNA concentrations were checked using a QuBit HS DNA assay (Invitrogen) prior to submission to the Génome Québec Centre D’Expertise et de Services (Montreal, Canada), who conducted library preparation and sequencing. Illumina MiSeq amplicon sequencing with 300 bp paired-end reads was conducted using the 16S rRNA region for bacteria with the V5-V6 fragment (forward primer 799F-mod3 CMGGATTAGATACCCKGG and reverse primer modified 1115R AGGGTTGCGCTCGTTG) as in McFrederick and Rehan (2019) and the ITS region for fungi with the ITS1 fragment (forward primer ITS1F CTTGGTCATTTAGAGGAAGTAA and reverse primer ITS2 GCTGCGTTCTTCATCGATGC).

Qiime2 was then used to process reads for microbiome analysis (Bolyen et al., 2019). Demultiplexed sequences underwent sequence quality control using the DADA2 pipeline, which filters phiX reads, chimeric sequences, and joins paired ends (Callahan et al., 2016). Sequences were omitted when quality scores dropped below 30 and read lengths fell below 283 bases for forward reads and 260 bases for reverse reads. Qiime2 was also used to generate feature tables, representative sequences, and taxonomy tables (Price et al., 2010; McDonald et al., 2012; Katoh and Standley, 2013; Weiss et al., 2017; Bolyen et al., 2019). ASVs were tested against the SILVA 128 99% OTUs full length sequences classifier for 16S bacterial sequences and the UNITE 99% OTUs classifier for ITS sequences using the q2-feature-classifier and classify-sklearn pipeline (Pedregosa et al., 2011; Yilmaz et al., 2014; Bokulich et al., 2018; Abarenkov et al., 2021). The SILVA database with 99% sequence identity was used for its refinement and removal of duplicate sequences (Glöckner et al., 2017). Taxonomic classifications were then cross referenced against the NCBI nt database using BLAST, where classifications from the NCBI database were used to clarify and prioritized when there were any discrepancies within the two classifiers (Johnson et al., 2008, 2021).

Resultant amplicon sequence variants (ASVs) read counts and taxonomic classification tables for each ASV were imported into R (version 3.6.1) for further statistical analysis (R Core Team, 2019). ASVs of the genera *Wolbachia* and *Sodalis* were removed as they are common intracellular endosymbionts present due to mite contamination (Graystock et al., 2017). While one blank did not contain any reads, ASVs identified in the other two of blanks were reagent or human-sourced contaminants and either absent in all samples or had low read counts of less than 50 reads. Using the “phyloseq” package, reads from three blanks were proportionally removed (McMurdie and Holmes, 2013).

Alpha and beta diversity analyses, measured using the Shannon diversity index and Bray–Curtis dissimilarity respectively, were conducted using the “phyloseq” package (McMurdie and Holmes, 2013). The adonis function was used to conduct permutational multivariate analyses (PERMANOVA) that test whether microbial composition varies significantly in the different levels of urbanization (Oksanen et al., 2020). Assumptions required for the PERMANOVA test were validated using the betadisper function and significant results followed up with Tukey’s HSD test (Oksanen et al., 2020).

Bipartite networks were created using the “bipartite” package in R, examining associations between the top 18 bacterial and fungal taxa and land use development gradients (Dormann et al., 2008, 2009; Dormann, 2011). Statistics were calculated at the species level, examining the degree of connectance, effective number of interacting partners, Shannon diversity of interactions, and closeness centrality in a weighted network across five categories of land use development (Dormann et al., 2008). Redundancy analyses (RDA) were conducted using the rda function from the “vegan” package (Oksanen et al., 2020). Using the decostand function in “vegan,” the Hellinger transformation was applied to taxa abundances and the environmental variables were standardized prior to RDA analyses (Legendre and Gallagher, 2001). An ANOVA like permutation test was performed with the anova.cca function to determine the significance of which environmental features could model microbe abundance.

Similarity percentage (SIMPER) values were calculated within the PAST (version 4.07) program to identify taxa predominantly leading to differences in diversity (Hammer et al., 2001). Furthermore, correlation analyses using CoNet and SparCC were conducted to find co-occurring bacterial and fungal taxa amongst all the bees. CoNet was performed using the package “CoNetinR” and edge scores calculated with Spearman, Bray, Pearson, and Kullback–Leibler (Faust and Raes, 2016). The package “SpiecEasi” was used to conduct SparCC analyses with 100 bootstrap replicates (Friedman and Alm, 2012).

## Results

Metabarcoding of the 58 adult *C. calcarata* resulted in an average of 31,394 reads, ranging from 19,860 to 43,593 paired-end reads per sample. The average quality of these reads was 34.5. A total of 192 bacterial and 367 fungal amplicon sequence variants (ASVs) with a mean sequence length of 317 bp were found and compared across 58 bee samples.

### Diversity

Microbial community composition did not reveal differences across urban land use gradients through alpha diversity or due to sample collection date over the range of 2 years (Supplementary Figure S1). Sample collection year did not associate with any differences in the alpha diversity, beta diversity, or relative abundance of bacterial and fungal taxa. Shannon diversity index comparisons across each environmental variable revealed no overall significant differences in microbial alpha diversity among the five categorical levels of developmental percent, green space percent, temperature, or precipitation (Supplementary Figure S1).

**Figure 2:**
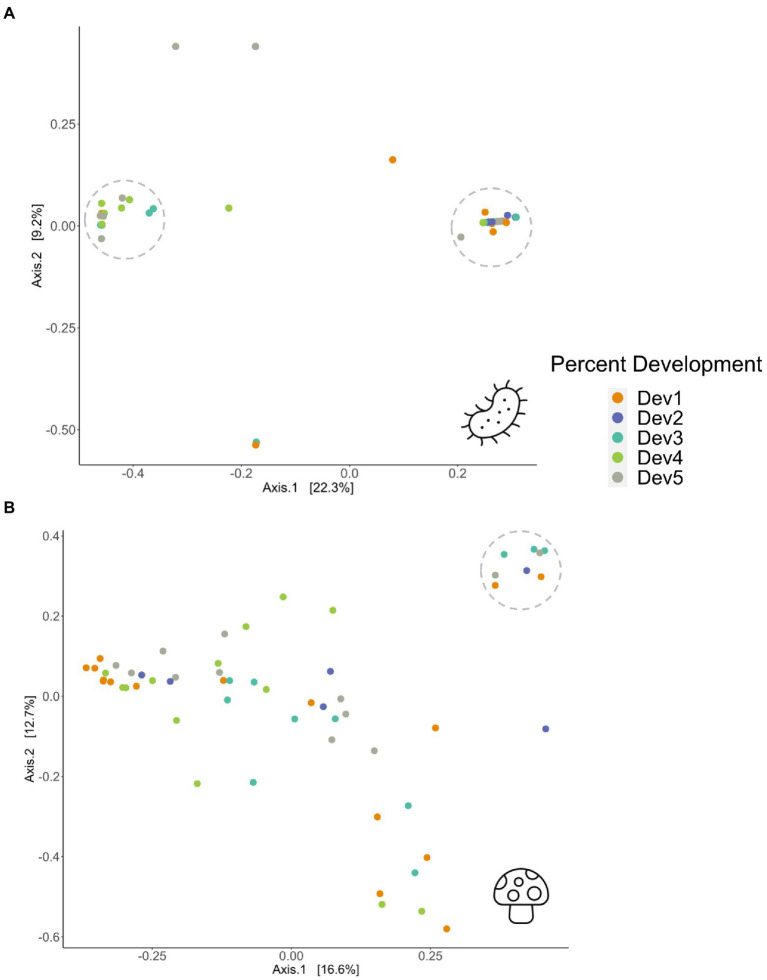
PCoA plots of Bray–Curtis dissimilarity matrices showing **(A)** bacterial (*p =* 0.017) and **(B)** fungal (*p* = 0.023) beta diversity in 58 adult *Ceratina calcarata* across five levels of percent development, ranging from Dev 1 (very low development) through Dev 5 (very high development). For exact development percentages, see Supplementary Table S1. Dotted circles represent clusters of individuals with similar beta diversity.

Bray-Curtis dissimilarities revealed bacterial and fungal differences in beta diversity across three environmental variables including land use development, precipitation and temperature (Figure 2; Supplementary Figure S2). Land use development percent was associated with both bacterial and fungal beta diversity (PERMANOVA; bacteria, *R*^2^ = 0.10, *df* = 4, *p =* 0.017; fungi, *R*^2^ = 0.10, *df* = 4, *p =* 0.023; Figure 2). Figure 2A indicates that individuals from moderate to very high levels of development were similar in microbial composition and dissimilar to individuals from sites with very low to low levels of development. Samples from sites with very high development had more bacterial genera richness than sites with low development (Supplementary Table S2), corroborating that development positively associates with bacterial richness. However, green space percent was not a significant factor in determining differences in Bray-Curtis diversity (PERMANOVA; bacteria, *R*^2^ = 0.07, *df* = 4, *p =* 0.18; fungi, *R*^2^ = 0.09, *df* = 4, *p =* 0.056; Supplementary Figures S2A,B).

Temperature explained variations in fungal beta diversity (PERMANOVA; fungi, *R*^2^ = 0.09, *df* = 4, *p =* 0.021; Supplementary Figure S2D), while bacterial beta diversity did not pass the test for homogeneity of multivariate dispersions with temperature (betadisper; bacteria, *F* = 3.13, *df* = 4, *p* = 0.018; fungi, *F* = 0.33, df = 4, *p* = 0.855; Supplementary Figure S2C). As for precipitation gradients, fungal beta diversity differences were detected, while bacterial beta diversity differences were insignificant (PERMANOVA; bacteria, *R*^2^ = 0.08, *df* = 4, *p =* 0.181; fungi, *R*^2^ = 0.12, *df* = 4, *p =* 0.002; Supplementary Figures S2E,F). Clear clustering was less evident for the fungal PCoA, suggesting increased dissimilarity between individuals (Figure 2B). One group of fungal samples that were clustered, indicating similar beta diversity, tended to have moderate to high annual temperatures, and low to moderate annual precipitation (Supplementary Figures S2D,F). This was also consistent comparing the average number of genera across the environmental variables, which saw that the low to moderate development, high temperature, and low precipitation categories had the highest fungal genera richness (Supplementary Table S3). However, the individuals grouped closely on the PCoA were spread across different levels of land use development and green space, suggesting the environmental variables are not always correlated with each other. As temperature, green space, and precipitation were not significant environmental variables for bacteria, this comparative analyses between the environmental variables could not be performed for the bacterial PCoA.

**Figure 3:**
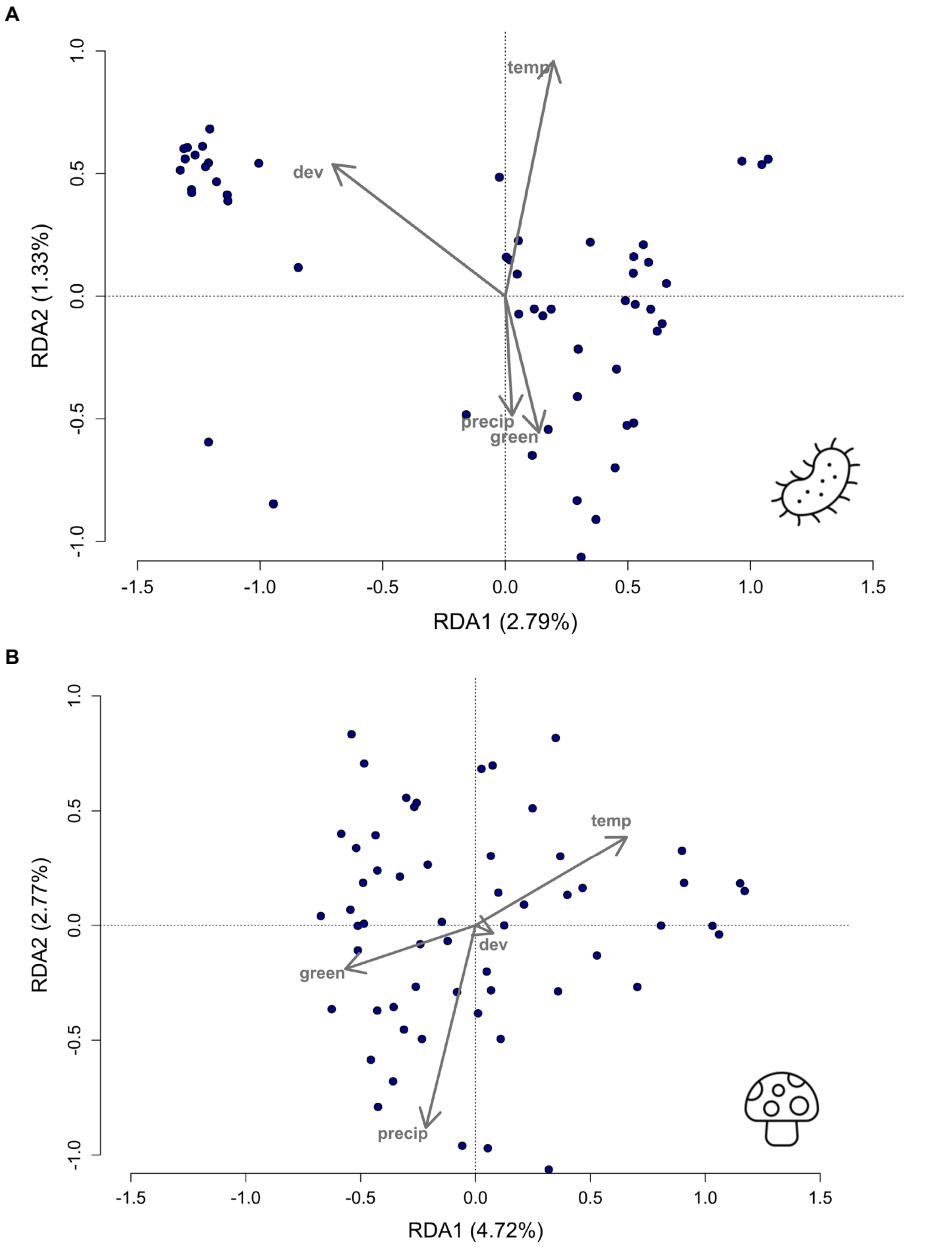
Redundancy analyses (RDA) plot showing whether **(A)** bacterial (*p* = 0.030) and **(B)** fungal (*p* = 0.001) taxa are associated with the environmental variables of dev = development, green = green space, temp = temperature, and precip = precipitation. Bacterial taxa are influenced by the development (*p =* 0.032) and green space (*p* = 0. 037) variables. Fungal taxa are driven by green space (*p =* 0.002) and precipitation (*p* = 0.012).

An RDA was conducted and analyzed using an ANOVA with 999 permutations on all four environmental variables to determine which variables were associated with bacterial and fungal taxa (Figure 3). Development was significant (ANOVA; bacteria, *F* = 1.86, *df* = 1, *p =* 0.032; Figure 3A) in associations between urbanization level and bacterial taxa. Green space was significantly associated with both bacterial and fungal taxa (ANOVA; bacteria, *F* = 1.86, *df* = 1, *p* = 0.037; fungi, *F* = 2.45, df = 1, *p* = 0.002; Figures 3A,B). Precipitation was also key in the RDA analyses for fungal taxa (ANOVA; fungi, *F* = 1.86, *df* = 1, *p =* 0.012; Figure 3B) with variation in precipitation explaining variation in fungal taxa. In addition to the RDA with all environmental variables, forward selection modelling was performed to select the driving environment variables that could predict diversity. Bacterial taxa revealed a significant model (ANOVA; bacteria, *F* = 1.80, *df* = 1, *p =* 0.016, adjusted *R*-squared = 0.017) associated with development (ANOVA; bacteria, *F* = 1.86, *df* = 1, *p =* 0.036) and temperature (ANOVA; bacteria, *F* = 1.74, *df* = 1, *p* = 0.046). The fungal model resulted in a different significant model (ANOVA; *F* = 1.97, *df* = 1, *p =* 0.007, adjusted *R*-squared value of 0.17) involving only temperature (ANOVA, fungi, *F* = 1.97, *df* = 1, *p* = 0.005) predicting fungal taxa.

### Taxonomy

**Figure 4:**
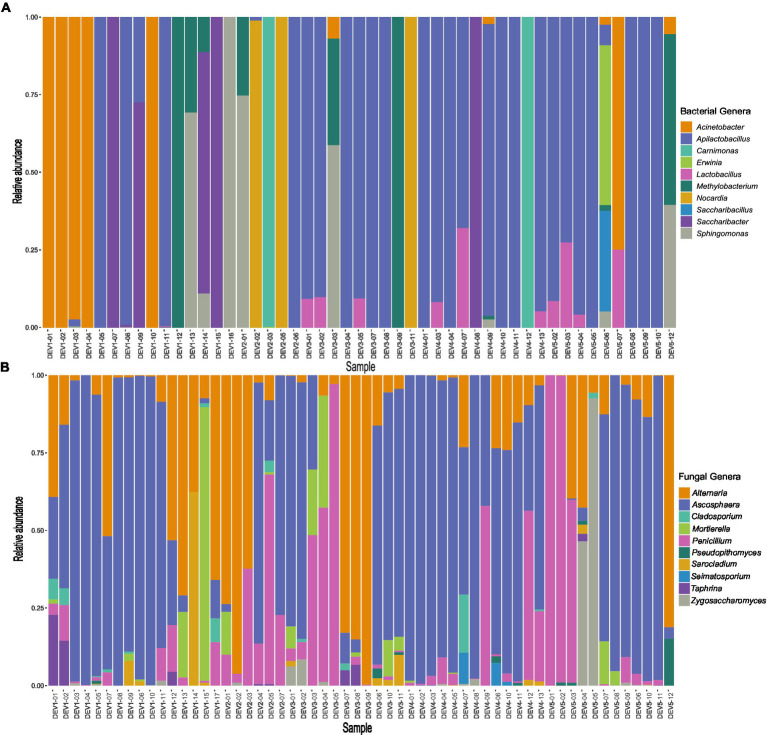
Top 10 **(A)** bacterial and **(B)** fungal genera found in 58 *Ceratina calcarata* from different levels of development across Toronto. The five categories range from Dev 1 (very low development) through Dev 5 (very high development).

Across the 58 samples, the bacterial genera *Acinetobacter, Apilactobacillus*, *Nocardia,* and *Saccharibacter* had the greatest summed relative abundances amongst all the bacterial genera (Supplementary Table S2). Particularly notable, *Apilactobacillus* had a relative abundance of over 50% of the total reads in 25 samples (Figure 4A). A low amount of bacterial diversity is noticed amongst the adults, as 26 samples contained reads from only one genus and 12 samples had two bacterial genera (Figure 4A; Supplementary Table S2). Overall, there was an average of 3.6 bacterial genera associated with each bee. In terms of fungi, *Alternaria, Ascosphaera*, and *Penicillium* had the greatest summed relative abundances and were common genera in the bee microbiome (Supplementary Table S3). Fungal genera richness was higher than bacterial, with an average of 6.5 genera per individual (Figure 4B; Supplementary Table S3).

Similarity percentage (SIMPER) analyses corroborated bacterial and fungal relative abundances were driven by environmental features (Supplementary Table S4). Some bacteria and fungi are typically overrepresented at either high levels of development or green space, suggesting patterns along an urbanization gradient (Supplementary Table S4). For example, *Acinetobacter* and *Saccharibacter* had high abundances in very low levels of development (Supplementary Table S4). On the contrary, *Lactobacillus* bacteria were found mostly in areas with moderate to high levels of development (Supplementary Table S4). *Apilactobacillus* was simultaneously overrepresented in areas with the highest amount of green space, least amount of development, and high levels of development (Supplementary Table S4). In terms of fungi, *Ascosphaera* was similarly abundant at both ends of the spectrum at low, high, and very high levels of development (Supplementary Table S4). *Taphrina* fungi were overrepresented in areas with very low levels of development, whereas the opposite was true for *Zygosaccharomyces* being overrepresented in areas of high development (Supplementary Table S4). Differences in taxa abundances were also apparent across varying precipitation and temperature gradients, with *Alternaria* being underrepresented with increased levels of precipitation and *Apilactobacillus* most abundant in environments with low annual temperature (Supplementary Table S4). In examining the clustered individuals on the fungal PCoA from sites with low precipitation and high temperatures (Supplementary Figures S2D,F), *Alternaria* was present in all these samples, with other common fungi including *Mortierella* and *Ascosphaera*. Thus, urbanization markedly characterizes disparate overrepresentations in bacteria and fungi.

To examine the uniqueness and connectedness of the microbiome across an urbanization gradient, bipartite networks were used to examine associations between different levels of development and microbial genera (Table 1; Supplementary Table S5; Supplementary Figure S3). The bacterial network resulted in overall lower levels of connectance (bacteria, 0.54; fungi, 0.82), links per species (bacteria, 2.13; fungi, 3.26), and Shannon diversity (bacteria, 1.56; fungi, 2.53) when compared to the fungal network. Bees from sites with the greatest percent development were found to have a higher degree of bacterial connectance, Shannon diversity of interactions, and effective partners, while also inversely showing low weighted closeness, when compared to areas with the least percent development (Table 1). Sites with low to moderate and very high percent development showed more abundant but less specialized relationships between bacteria and bees. This pattern held for fungi at a moderate level of land use development (Table 1). Thus, fungal networks maintained more consistency across the urban land use gradients. Networks revealed certain microbes like *Apilactobacillus, Alternaria, Ascosphaera*, and *Penicillium* had high degrees of connectance across all five development levels, whereas others had low degrees of connectance and were associated with an urbanization level. For example, *Clostridium* and *Saccharibacillus* were only found in very high levels of development and *Enterobacter* and *Samsoniella* were associated with very low levels of development.

**Table 1:** Summary of bipartite network level statistics comparing the association between the top 18 bacterial and fungal taxa across five levels of development.

	Network level statistics	Dev 1	Dev 2	Dev 3	Dev 4	Dev 5
Bacteria	Degree	8	6	10	10	15
	Effective partners	1.45	2.15	2.11	1.06	2.33
	Shannon diversity	0.34	0.77	0.75	0.06	0.85
	Weighted closeness	0.55	0.03	0.06	0.51	0.12
Fungi	Degree	14	14	17	14	16
	Effective partners	1.97	3.43	4.50	2.07	3.80
	Shannon diversity	0.68	1.23	1.50	0.73	1.33
	Weighted closeness	0.35	0.08	0.19	0.34	0.28

The five categories range from Dev 1 (very low development) through Dev 5 (very high development).

### Bacterial and fungal co-occurrences

CoNet and SparCC analyses were used to determine if any bacterial and fungal associations were found among the top 10 bacterial and fungal taxa across all 58 samples (Table 2; Supplementary Tables S6, S7). Using all individuals, CoNet revealed 17 associations (Supplementary Table S6), while SparCC presented 28 total co-occurrences of positive and negative correlations (Supplementary Table S7). Interestingly, a positive association between bacteria-bacteria was found to be significant across both statistical analyses in *Sphingomonas* and *Saccharibacillus* (CoNet, correlation = 0.31, *p* = 0.019; SparCC, correlation = 0.53, *p* < 0.01; Supplementary Tables S6, S7). Additionally, separate analyses for each land use development level revealed patterns in co-occurrences. Through CoNet analyses, *Metholybacterium* was only found not correlated to *Sphingomonas* in the very low and moderate development levels, while *Acinetobacter* and *Sphingomonas* were associated only in the moderate to high development levels (Table 2). Associations between fungi-bacteria and fungi-fungi were absent in densely urban areas and only seen in sites with the lowest land use development, with the rest of the associations only existing between bacteria (Table 2).

**Table 2:** CoNet correlations between bacteria-bacteria, fungi-fungi, and fungi-bacteria within the top 10 bacterial and fungal genera found in 58 *Ceratina calcarata* across five levels of land use development.

Development level	Correlated taxa	Correlation	*p*-value
DEV-1 (*n* = 16)	*Alternaria – Penicillium*	0.81	0.013
	*Sarocladium – Saccharibacter*	0.75	0.015
DEV-2 (*n* = 6)	*Carnimonas – Erwinia*	1.00	<0.01
	*Methylobacterium – Sphingomonas*	1.00	<0.01
DEV-3 (*n* = 11)	*Acinetobacter – Sphingomonas*	1.00	<0.01
DEV-4 (*n* = 13)	*Acinetobacter – Methylobacterium*	1.00	<0.01
	*Acinetobacter – Sphingomonas*	1.00	<0.01
	*Methylobacterium – Sphingomonas*	1.00	<0.01
DEV-5 (*n* = 12)	*Acinetobacter – Saccharibacillus*	1.00	<0.01
	*Acinetobacter – Erwinia*	1.00	<0.01
	*Erwinia – Saccharibacillus*	1.00	<0.01
	*Methylobacterium – Sphingomonas*	0.98	<0.01

The five categories range from Dev 1 (very low development) through Dev 5 (very high development). Sample sizes at each land use development level are provided in brackets. A full representation of all correlations is available in the supplement (Supplementary Table S6).

## Discussion

This study examines the bacterial and fungal microbiome in 58 *C. calcarata* individuals across an urban land use gradient. Percent development, percent green space, annual temperature, and annual precipitation were examined to determine how environmental factors may drive differences in microbial diversity and composition. While alpha diversity did not differ across the city, beta diversity and redundancy analysis modelling could be predicted by percent development and temperature. Taxonomic comparisons also revealed some bacterial and fungal taxa were more commonly found in either very low or highly developed areas of the city, indicating differences in the microbiome between urban land use gradients. Different levels of land use development also result in varying degrees of connectance in networks and different co-occurrences between microbes.

### Microbial diversity

Shannon’s diversity indices, a measure of alpha diversity, did not vary with environmental variables of development, green space, precipitation, or temperature and yielded low values of bacterial diversity, where more than half of the samples only contained one or two genera (Supplementary Figure S1). This aligns with a previous study of *C. calcarata* in Toronto, which found little change and overall low microbial alpha diversity as bees matured from brood to adults (Nguyen and Rehan, 2022). Similarly, a study comparing the stingless bee *Tetragonula carbonaria* microbiome between two different sites also showed alpha diversity remaining consistent, despite climatic and floral resource differences (Hall et al., 2021). Although studies with stingless bees have revealed the presence of environmental bacteria in the microbiome (Kwong et al., 2017b; Cerqueira et al., 2021), social bees often have a core microbiota and low diversity (Kwong and Moran, 2016). However, this is not representative of solitary wild bees, such as in a study with *Osmia lignaria* across different environmental contexts, which found that environmental factors drove differences in relative ASV abundances and alpha diversity (Cohen et al., 2020). Yet, as many factors affect microbiome alpha diversity it remains difficult to segregate how factors are affecting overall diversity in isolation and how a combination of environmental or situational variables co-occurring can affect alpha diversity.

Beta diversity, represented by Bray–Curtis dissimilarity matrices (Figure 2), was able to capture more of the microbial differences driven by urban land use gradients. Percent development was the most significant factor, with development associating with both bacterial and fungal Bray-Curtis dissimilarities, high development showing increased bacterial genera richness, and with development able to predict bacterial diversity through the redundancy modelling analyses (Figures 2, 3; Supplementary Table S2). Annual temperature was another considerable variable, yielding a significant ability to model differences in both bacterial and fungal microbiomes and associate with fungal beta diversity (Supplementary Figure S2F). Collectively, the interplay between these environmental characteristics may be dynamically changing microbial diversity, as features such as high temperature and low precipitation can act together to foster higher fungal genera richness and clustered Bray-Curtis dissimilarities, as seen in the circled individuals on the PCoA plots (Supplementary Table S3; Supplementary Figures S2D,F). However, these samples did not cluster with either land use development or green space, suggesting that the correlation between environmental variables is unclear (Figure 2; Supplementary Figure S2). Environmental features were expected to be a factor determining differences in beta diversity, as was initially seen in a study comparing two colonies of stingless bees at different locations (Hall et al., 2021), and in *O. lignaria* when dissimilarity matrices could be predicted by percent natural cover, number of trees and shrubs, bee species richness, and bare soil (Cohen et al., 2020). Similarly, McFrederick and Rehan (2019) found different species richness of fungi and bacteria when comparing subtropical, temperate and grassland zones across Australia, suggesting that climate shapes the *C. australensis* microbiome. Thus, environmental characteristics describing both land use and climate affect the microbial diversity of individual *C. calcarata* microbiomes.

### Microbial composition

*Apilactobacillus*, *Alternaria*, *Penicillium* and *Ascosphaera* were the most prevalent and abundant bacterial and fungal genera found across the city (Supplementary Tables S2, S3). *Apilactobacillus* are common beneficial bee symbionts (Tlais et al., 2022) and were established as part of the core microbiome in *C. calcarata* in New Hampshire, a more rural landscape (McFrederick and Rehan, 2016; Graystock et al., 2017). In urban cities such as Toronto, *Apilactobacillus* was previously largely absent in adult *C. calcarata* (Nguyen and Rehan, 2022) and was found to be underrepresented at sites with moderate levels of land use development, overrepresented in sites with the most green space, and overrepresented at sites with lower annual temperatures in this study (Supplementary Table S4). Thus, urban bees reveal a different microbiome from those in rural contexts and of particular concern is the varying abundance of *Apilactobacillus*.

The fungal genus *Ascosphaera* contains both pathogenic and apathogenic fungi (Klinger et al., 2013), and the species *A. major* was common in *C. calcarata* (Supplementary Table S3). This species has caused chalkbrood-like diseases in *Megachile centuncularis* and *Apis mellifera*, but can also live relatively harmlessly as a facultative parasite within bee nests on pollen provisions and larval feces, including other wild bees such as *Osmia bicornis* (Holm and Skou, 1972; Bissett, 1988; Wynns et al., 2013). Therefore, the abundance of *Ascosphaera* may indicate a commensalism between *C. calcarata* and these bee specialist fungi. Future studies are needed to determine the fitness effects of *Ascosphaera* on this species.

Overrepresentations of certain bacterial and fungal taxa at sites of varying land use development may indicate such factors are affecting microbial composition. Areas with a low percentage of development were found to have a greater abundance of *Acinetobacter, Ascosphaera, Saccharibacter,* and *Taphrina* (Supplementary Table S4). *Acinetobacter* is a flower-associated species of bacteria also commonly associated with yeasts in nectar which can induce germination and pollen bursting that then benefits pollinators by way of improved nutrition from nectar (Christensen et al., 2021; Rering et al., 2021). Another flower-associated bacteria, *Saccharibacter*, is closely related to the bacteria *Bombella apis* which is known to protect developing honey bees from fungal pathogens and contains genetic loci involved with nutrition, microbial and host interactions, and immunity (Smith and Newton, 2020). Thus, the overrepresentation of beneficial microbes in areas with low land use development is promising for these pollinators. On the contrary, the two fungal genera found in high abundance in more rural areas, *Ascosphaera* and *Taphrina,* are facultative bee and plant pathogens, respectively, (Cissé et al., 2013; Wynns et al., 2013). However, these genera were also previously seen in immature *C. calcarata* and it is unclear if they pose any threat to this species (Nguyen and Rehan, 2022). *Ascosphaera* was also overrepresented at high and very high development levels (Supplementary Table S4; Figure 4B), suggesting this fungi may not be limited to rural areas.

The overrepresented genera present in sites with a high percentage of development, such as *Lactobacillus*, *Penicillium,* and *Zygosaccharomyces* (Supplementary Table S4), were not microbes that are known to be harmful to bee health. *Lactobacillus* spp., such as *L. crispatus* and *L. intestinalis*, have been seen in *A. mellifera*, *Bombus terrestris*, and *O. bicornis* (Mohr and Tebbe, 2006), and many studies have uncovered the important and beneficial relationship between *Lactobacillus* and bees (Rothman et al., 2019; Li et al., 2021). *Penicillium* molds are commonly found in *Melipona scutellaris* (Barbosa et al., 2018) and *A. mellifera* bee bread and is of importance as it produces enzymes involved in lipid, protein, and carbohydrate metabolism that can even protect bees against fungicides (Gilliam et al., 1989; Yoder et al., 2013). *Zygosaccharomyces* sp. are fungi that have been shown to provide steroid precursors crucial for the pupation of the stingless bee *Scaptotrigona depilis* (Paludo et al., 2018). Hence, while differing from areas of low land use development, bacteria and fungi found in urban bees may be supported by their own beneficial properties to their bee hosts. Regardless of urbanization, different overrepresentations of bacteria and fungi may serve varying, but equally beneficial, purposes. Areas with low development seem to harbour plant associated microbes that may be associated with natural plant availability, whereas high development sites contain microbes more associated with bee development and digestion.

Co-occurrences between microbes have been studied in bees and pollen to examine how microbial members interact and establish the microbiome (Russell et al., 2011; Graystock et al., 2017; Manirajan et al., 2018; Dew et al., 2020). This study found a strong positive relationship between *Sphingomonas* and *Saccharibacillus* when examining adults (Supplementary Tables S6, S7). While *Saccharibacillus* has been found in commercial bee pollen from Europe, little is known about its interactions and presence in bee microbiomes (Andrade et al., 2019). Bacteria of the genus *Sphingomonas* have been shown to be negatively correlated with *Fusarium* species that cause Fusarium Head Blight in maize crops (Cobo-Díaz et al., 2019). In *C. calcarata*, *Sphingomonas* co-occurred positively with the fungal genera *Pantoea* (Nguyen and Rehan, 2022), a genus prevalent in *C. australensis* (Shell and Rehan, 2022), *A. mellifera* (Wright et al., 2001), and stingless bees (Leonhardt and Kaltenpoth, 2014). Additionally, *Sphingomonas* is a dominant bacteria found in the nests of stingless bees *Frieseomelitta varia*, *Melipona quadrifasciata*, and *Tetragonisca angustula* (de Sousa, 2021) and is also found in *A. mellifera* (Anjum et al., 2018; Muñoz-Colmenero et al., 2020) and *O*. *bicornis* microbiomes (Mohr and Tebbe, 2006), thus this bacteria co-occurs naturally with wild bees. In regards to urban and agricultural bees, this bacteria may be particularly beneficial as it contains enzymes that degrade organochlorides in insecticides (Russell et al., 2011). Therefore, commonly occurring bacteria, such as *Sphingomonas*, may be playing an underappreciated role in the wild bee microbiome.

Microbe correlations can be examined considering land use development to determine if environmental factors may be affecting the stability of these associations. *Methylobacterium* was correlated with *Sphingomonas* in all but the low and moderate percent development sites (Table 2). *Methylobacterium* have shown beneficial relationships with plants and bacteria, sometimes even relying on growth factors produced by other microbes (Iguchi et al., 2015; Manirajan et al., 2018). The number of correlations present also increased with percent developed area, suggesting that urbanized areas may be associated with more positive co-occurrences between bacteria. This has been seen in urban soils, where environmental features altered microbial networks (Wang et al., 2018). Therefore, landscape features may be changing the way bacteria and fungi are supported, which can in turn affect the presence and abundance of these and other microbes. Further examination into the functional role of specific microbes as well as how they exist in symbioses is needed to explain how these networks are maintained.

### Effects of a changing environment

Changes in microbial diversity and composition are of potential concern because bee-microbe symbioses play a key role maintaining bee health (Engel et al., 2012; Mockler et al., 2018; Dharampal et al., 2019, 2020; Rothman et al., 2019). While this study described several abundant bacterial and fungal genera dominating the microbiome, this is a large contrast to previous studies of *C. calcarata* that revealed 13 core bacterial phylotypes in bees and much more diversity (Graystock et al., 2017). The reason for bacteria showing decreased diversity compared to fungi, as seen in lower Shannon diversity measures, lower degrees of network connectance, and fewer effective partners (Table 2), remains unclear. This low bacterial diversity in adults was also found in a previous study of *C. calcarata* in Toronto, particularly when compared to developing brood, and suggests a persistent and concerning decrease in microbial diversity in urban landscapes (Nguyen and Rehan, 2022). Ongoing long-term and additional studies are needed to examine whether the few bacterial genera currently making up the microbiome are excluding other bacteria and/or whether bees in cities have generally less diverse microbiomes in this and other wild bee species.

As the urban land use gradients such as percent development and temperature reveal their effects on the bee microbiome, rapid urbanization becomes increasingly alarming. Urbanization and anthropogenic activities are a worsening problem driving declines in bee populations, altering bee community compositions, and negatively affecting certain bee species (Ritchie and Roser, 2018; Wilson and Jamieson, 2019; Ayers and Rehan, 2021; Prendergast et al., 2022). In addition, the presence of pesticides in urban areas may be driving declines in bee health and in bee microbiome structure, composition, and diversity (Kakumanu et al., 2016; Botías et al., 2017; Hotchkiss et al., 2022). Future work comparing a wider range of rural and agricultural landscapes across multiple regions will help determine how bee microbiomes change with land use. Additional studies examining pesticides present in bee habitats in urban and rural areas and how these accumulate in bees and their pollen provisions will also be important (Pisa et al., 2014; Kakumanu et al., 2016; Botías et al., 2017; Hotchkiss et al., 2022). As research continues to untangle the variables that work together to establish and maintain the wild bee microbiome, the ever-changing landscape in cities adds new considerations for possible environmental stressors.

In conclusion, this study examines the bacterial and fungal composition and diversity in adult *C. calcarata* across an urban land use gradient, revealing differences explained by percent land use development, green space, precipitation, and temperature. Individuals from low to moderate development levels tended to share similar bacterial composition within one cluster, while those from moderate to high development levels grouped separately. In examining fungal taxa, individuals showed greater dissimilarity in beta diversity, with climatic variables as possible drivers. The interplay of environmental factors across urbanization gradients act on microbial community composition, with overlapping characteristics such as annual temperature and annual precipitation coinciding with fungal beta diversity. Microbial composition in rural areas were dominated by genera such as *Acinetobacter* and *Apilactobacillus*, beneficial microbes that support bee health and may affect bee survival. Specific taxa varied across different levels of urbanization, which may be explained by co-occurrences between bacteria and fungi that varied amongst different land use development, and suggests that microbial relationships are dependent on changes in environment. These complex networks reveal that urban areas may exhibit a stronger degree of connectance in bacteria, while lower levels of urbanization foster greater connectance within the fungal microbiome. Overall, increased urbanization has led to a significant impact on microbial composition and diversity. As cities continue to expand and urbanization rises globally, it is increasingly important to understand how landscapes affect bee health through their microbiome.

## Data availability statement

The datasets presented in this study can be found in online repositories. The names of the repository/repositories and accession number(s) can be found at: https://www.ncbi.nlm.nih.gov/ BioProject: PRJNA805022 BioSamples: SAMN25891990-SAMN25892379.

## Author contributions

PN: sample preparation, DNA extraction, bioinformatics, and analyzed and visualized data. SR: conceived study, supervised, and provided funding. PN and SR: wrote and approved final manuscript. All authors contributed to the article and approved the submitted version.

## Funding

This study was funded by the Weston Family Foundation Microbiome Initiative, NSERC Discovery Grant, Supplement and E.W.R. Steacie Fellowship to SR.

## Conflict of interest

The authors declare that the research was conducted in the absence of any commercial or financial relationships that could be construed as a potential conflict of interest.

## Publisher’s note

All claims expressed in this article are solely those of the authors and do not necessarily represent those of their affiliated organizations, or those of the publisher, the editors and the reviewers. Any product that may be evaluated in this article, or claim that may be made by its manufacturer, is not guaranteed or endorsed by the publisher.
