# Supplementary material for: The effects of urban land use gradients on wild bee microbiomes
Source: Front Microbiol. 2022 Nov 17;13:992660. doi: 10.3389/fmicb.2022.992660 (PMC9714450; doi:10.3389/fmicb.2022.992660)
Supplement: Supplementary file 2 [file Image_1.pdf]

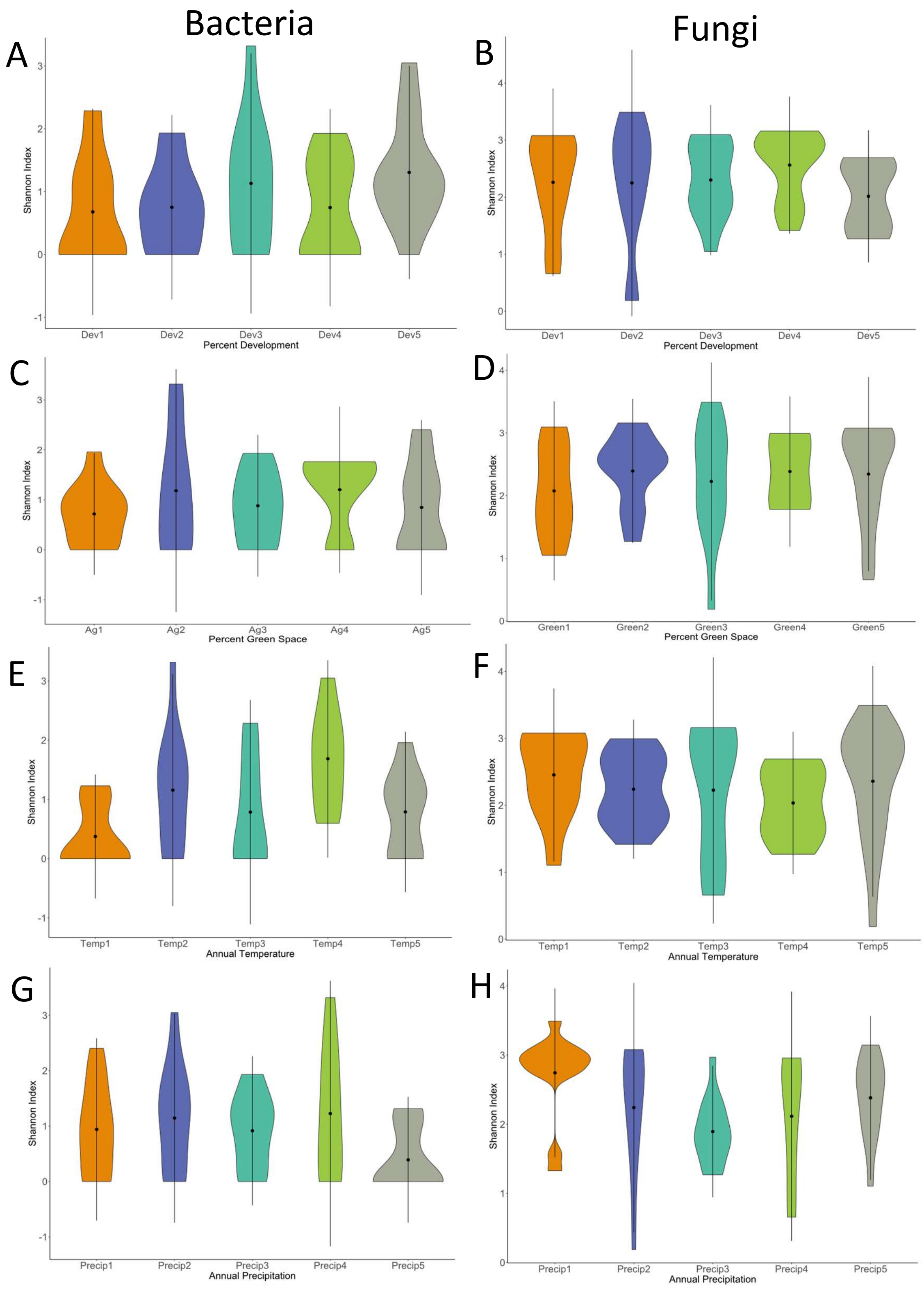


**Figure S1.** Violin plots depicting Shannon diversity of bacteria (left) and fungal (right) taxa across four environmental variables: (a-b) development, (c-d) green space, (e-f) temperature, and (g-h) precipitation. Exact categorical divisions can be found in Table S1. Vertical lines indicate the range of Shannon indices and the shape widths show distribution of samples.

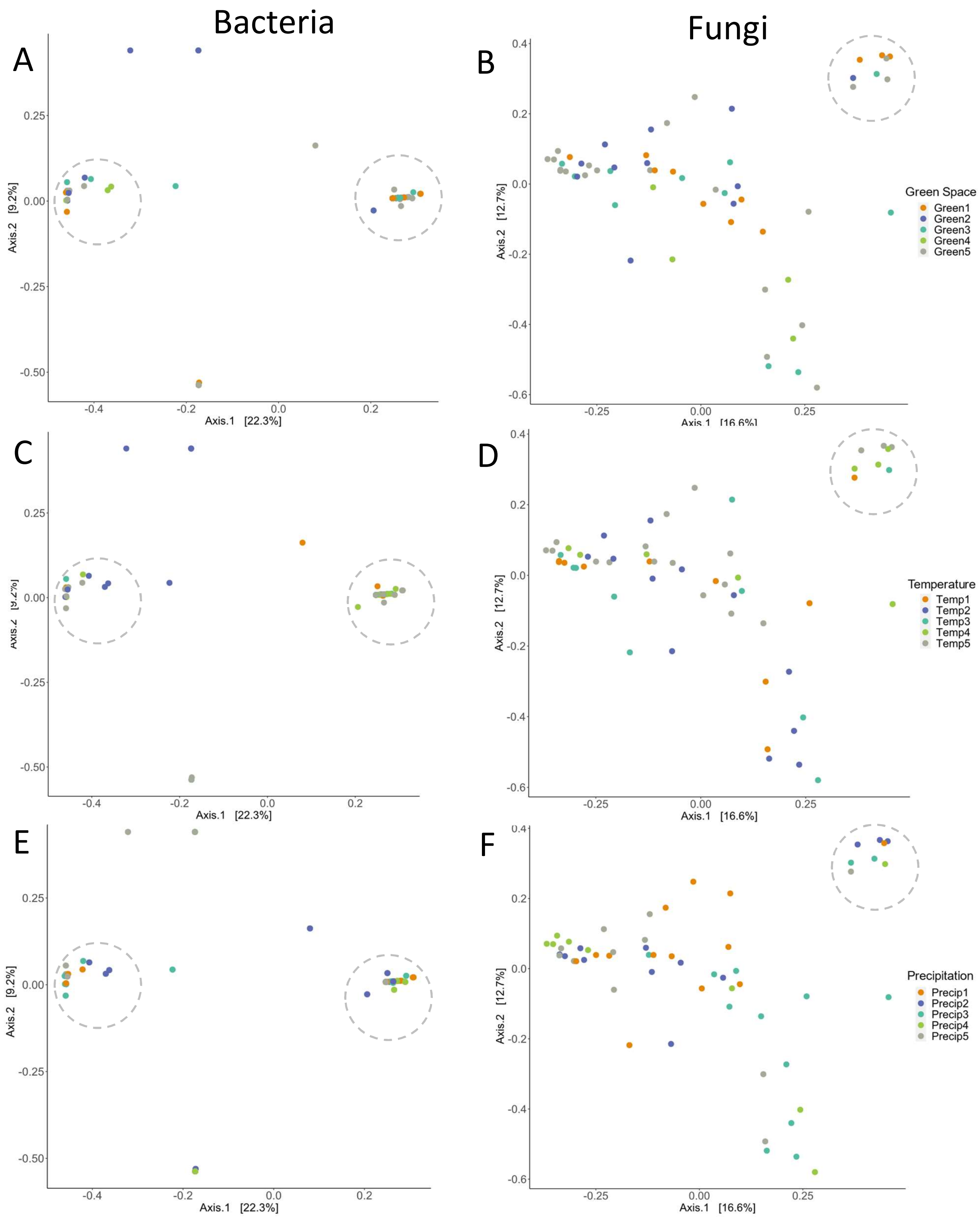


**Figure S2.** PCoA plots of Bray-Curtis dissimilarity matrices showing bacterial (left) and fungal (right) beta diversity when considering the variables of (a-b) green space (bacteria, $p = 0.18$; fungi, $p = 0.056$), (c-d) temperature (bacteria, $p = 0.013$; fungi, $p = 0.021$), and (e-f) precipitation (bacteria, $p = 0.181$; fungi, $p = 0.002$). Each category ranges from 1 (very low) to 5 (very high). Exact categorical divisions can be found in Table S1. Dotted circles represent clustered individuals with similar beta diversity.

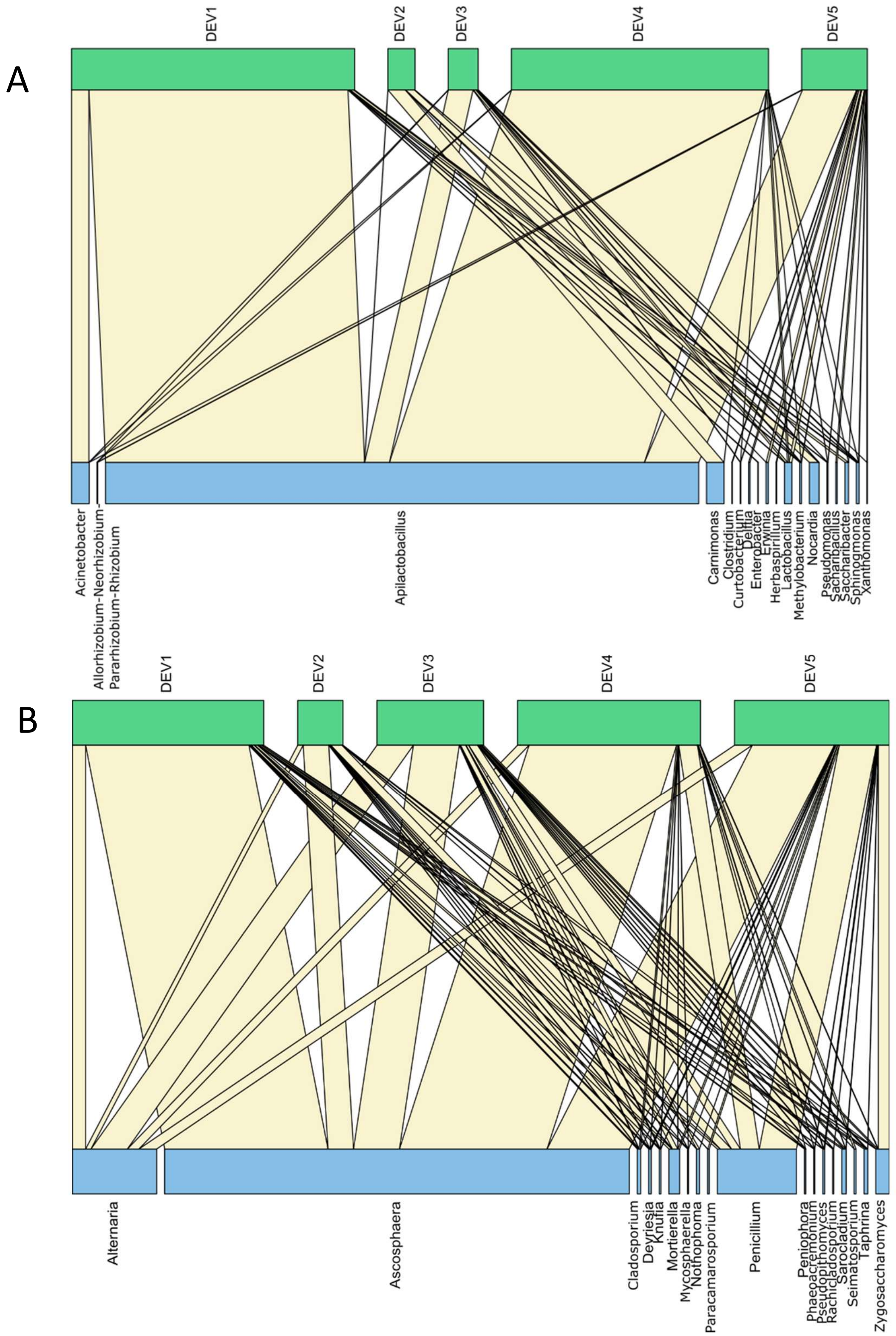


**Figure S3.** Bipartite networks comparing the association between the top 20 (a) bacterial and (b) fungal taxa and five levels of development. The five categories range from Dev 1 (very low development) through Dev 5 (very high development).
